# 

**Published:** 1781-08

**Authors:** 


### PROMOTIONS.

*July* 14. James Hervey, M. B. of Queen's College, Oxford, to be M. D.—29. Dr. Bree to be phyſician to the County Hoſpital at Northampton, in the room of Dr. Fothergill, who has reſigned.—31. Mr. John Connor to be ſurgeon to the General Hoſpital in the Leeward Iſlands.—*Auguſt* 1. William Hawes, M. D. to be phyſician to the Surry Diſpenſary.

---

### DEATHS.

Lately, Mr. Robert Chetham, ſurgeon and apothecary at Wakefield in Yorkſhire.—*June* 6. At Kingſton, Jamaica, Mr. Thomas Hamilton, late ſurgeon of the ſhip Minerva.—*July* 8. In the convent of the Feuillans at Paris, Friar John de Badillac, better known by the name of Frere Coſme, famous for his invention of the *Lithotome caché*, a deſcription of which firſt appeared in the *Journal des Sçavans* for 1748—14. At Portſmouth mouth Common, after a short illness, Mr. Jones, apothecary.—25. At Lewisham in Kent, Mr. Thomas M'Carthy, surgeon.——*August* 4. At Stamford in Lincolnshire, of a putrid sore throat, Mr. Gilbert Smith, surgeon and apothecary at Friestone near Grantham.

---

### SECTION IV.

### MONTHLY CATALOGUE.

1. AN account of a method of preserving water, at sea, from putrefaction, and of restoring to the water its original pleasantness and purity, by a cheap and easy process: to which is added a mode of impregnating water, in large quantities, with fixed air, for medicinal uses, on board ships, and in hospitals; and likewise a process for the preparation of artificial yeast. By *Thomas Henry*, F. R. S. and member of the Medical Society of London. 8vo. *Johnson*, London, 1781. 43 pages, with three copper plates.

A method of preserving water free from putrefaction was some years since proposed by the late Dr. Alston. It consisted in adding a quantity of lime to every cask of water. That substance is known to have a strong antiseptic property; perty; and water, as long as it retains the impregnation of lime, never putrefies. But the lime communicates a difagreeable tafte to the water, and, abftracted from that inconvenience, might, in many inftances, be detrimental.

To free the water, at the time of ufing it, from the lime, Dr. Alfton propofed the precipitation of the latter, by throwing into the water a quantity of magnefia alba; on this principle, that as lime ftone is rendered foluble in water by its deprivation of fixed air, and has a greater affinity with that air than magnefia has, the particles of quick lime diffolved in the water would attract the air from the magnefia, and thereby becoming no longer foluble, would fall to the bottom, and leave the water taftelefs and fit for œconomical ufes.

Dr. Alfton's theory was well founded, but the expence attending his method, owing to the price of magnefia, prevented it from being adopted. The procefs recommended in the pamphlet before us is free from this objection, and at the fame time is eafily practicable.

Mr. Henry's method of impregnating water with lime, confifts in adding two pounds of well-burnt quick lime to every cafk of water of 120 gallons. When the lime has been in the cafk fome

ſome minutes, and the heat and effervefcence occafioned by the mixture are over, the cafk is to be carefully ftopped from any communication with the external air. In order to reftore water thus impregnated to its original tafte the lime is to be precipitated by means of fixed air. For this purpofe our author recommends a cafk of a particular conftruction, of which an engraving is given in the work. The top is directed to be formed of one plank, and to have a piece cut out of the center, of a circular form, and as large as can be allowed without weakening the fides too much. This piece or bung is to be made to fit it as clofely as poffible, and to have an iron handle affixed to it, for the purpofe of lifting it, and of confining a weight which is to be laid on, to keep the bung from yielding to a fmall force from within. A fmall hole is likewife to be bored in the fide of the top, which is to be exactly ftopped with a plug.

A cafk thus conftructed, which may be fuppofed to contain fixty gallons, is to be fecured on a convenient part of the deck, or flung up in the fhrouds, and filled with lime water, drawn off clear from the fediment, fo as to avoid any vifible particles of lime floating in it; allowing fufficient room for the air veffel, and a free fpace

 of of about half an inch between the surface of the water and the top of the cask.

To complete the apparatus a vessel is also to be prepared, capable of containing two gallons, or $\frac{1}{30}$ of the capacity of the cask. Into this vessel are to be introduced half a pound of any mild calcareous earth (as marble, unburnt lime, stone, or chalk) grossly powdered, and two quarts of water. Three ounces of strong vitriolic acid are then to be poured gradually on this mixture, and a tubulated stopper placed in the mouth of the vessel, which is now to be let down by means of the strings into the cask. The fixed air, let loose from the mild calcareous earth, will bubble up through the lime water. When this has continued about a minute, the bung is to be fastened on, and a weight placed over the bung to keep it in its place. In about an hour the bung may be removed, in order to see whether the discharge of air continues. If it has ceased, or is considerably abated, three ounces more of vitriolic acid are to be added, and the air vessel returned to its former station in the cask; and, if the first parcel of calcareous earth and vitriolic acid be unequal to the sweetening of the lime water, they are to be removed, and a fresh quantity substituted in their place. During this this procefs, which, we are told, is generally completed in a few hours, the fmall plug is directed to be occafionally removed, in order to let out the part of the air which is not foluble in water.

After adding feveral cautions relative to the choice and prefervation of the quick lime, the keeping the cafks clean, and other circumftances neceffary to be attended to in thefe proceffes, our author proceeds to defcribe a method of impregnating water in large quantities with fixed air. For this purpofe a funnel fimilar to that ufed in Dr. Nooth's apparatus is to be cemented into the head of a cafk. A bent tube is likewife to be introduced through another part of the head and properly cemented. One end of this tube is to reach within a few inches of the bottom of the cafk, while the other extremity, by the intervention of a bladder, communicates with the lower part of Dr. Nooth's apparatus, containing the mixture of calcareous earth and vitriolic acid.

Mr. Henry's procefs for making artificial yeaft confifts in boiling flour and water to the confiftence of treacle, and when the mixture is cold impregnating it with fixed air; after which it is to be placed in a degree of heat between 70° and 80° of Fahrenheit's thermometer, and

ſtirred twice or thrice in twenty-four hours, by which means, in about two days, ſuch a degree of fermentation will have taken place, as to give the mixture the appearance of yeaſt. A quart of this yeaſt is directed to be mixed with ſix pounds of flour, kneaded with a ſufficient quantity of water. The dough is afterwards to ſtand twelve hours, or till it appears to be ſufficiently fermented, in a degree of warmth equal to that above-mentioned, and is then to be formed into loaves, and baked.

The author next deſcribes proceſſes for making artificial Pyrmont and Seltzer waters, which are ſimilar to thoſe mentioned by Sir T. Bergman (ſee our firſt volume, page 78). He likewiſe adds directions how to prepare Mr. Bewley's Julep, which is done by diſſolving three drachms of foſſil alkali in each quart of water, and throwing in ſtreams of fixed air, till the alkaline taſte is deſtroyed, and the water has acquired an agreeable pungency.

In a poſtſcript to the work Mr. Henry endeavours to remove ſome objections that have been made to the practicability of his plan. The principal of theſe was ſuggeſted to him by Dr. Lind, who doubted, that in a rough ſea the agitation of the ſhip would obſtruct the proceſs. In order to obviate this difficulty the author has defcribed a mode of fufpending the purifying cafk in fuch a manner, that it may always retain its level, whatever be the motion of the fhip.

2. *Edvardi Sandifort*, medicinæ, anatomes, et chirurgiæ, in academiâ Batava, quæ Leidæ eft, profefforis, Tabulæ inteftini duodeni. 4to. Lugduni Batavorum, 1780.

Thefe anatomical plates, which are five in number, are executed with great elegance and accuracy. The differtation prefixed to them is divided into three chapters. In the firft chapter the author gives a general defcription of the fituation and courfe of the duodenum; in the fecond he defcribes the preparations from which the drawings for this work were made; and the third contains an explanation of the feveral figures.

3. Differtatio inauguralis de genefi materiarum febres inflammatorias et lentas excitantium; auctore *Philippo Adolpho Fries*, Naffovia Siegenenfi, anatom. chirurg. artis obftetriciæ prof. publ. Monafterienfi, &c. 4to. Hardervici, 1780. p. 12.

4. Differtatio chemica de diverfa phlogifti quantitate in metallis, quam prefide Mag. Torb. Bergman, chem. prof. &c. publice ventilandam dam fiftit *And. Nicol. Tunborg*, Dalecarlo. 4to. Upfaliæ, 1780. p. 16.

This ingenious differtation is to be confidered as the work of profeffor Bergman. From the experiments related in it, it appears, that of all the metallic fubftances platina abounds moft with phlogifton; and that after platina the other metals rank, in this refpect, in the following order, viz. gold, copper, cobalt, iron, manganefe, zinc, nickel, antimony, tin, arfenic, filver, mercury, bifmuth, and lead.

5. Pharmacopœia Genevenfis, ad ufum Nofocomiorum. Auctoribus *Dan. de la Roche*, *Ludov. Odier*, et *Carol. Dunant*, M. D. D. et Soc. Med. Edinenf. Sociis. 8vo. Geneva, 1780. p. 193.

This is a judicious compilation from the beft works of this kind that have been publifhed of late in different parts of Europe.

6. Nachricht von zwey neu entdekten mitteln für fchwangere und gebæhrende; *i. e.* An account of two remedies lately difcovered for pregnant and lying-in-women. 8vo. Berne, 1780. 109 pages.

This is the production of a dealer in noftrums.

7. Vorfchlæge an Mütter welche ihre kinder felbft zu ftillen gedenken, &c. *i. e.* Advice to thofe mothers who fuckle their children. By

Madame

Madame *le Rebours.* Tranflated from the French, with additional remarks, by J. E. S. 8vo. Bafil, 1780.

A judicious performance. This tranflation was undertaken by Profeffor Simon, at the requeft of the *Philanthropical Society* at Strafburgh. The notes are by his friend Dr. Schweighæufer.

8. Differtatio medica inauguralis de morbis cutaneis. Auctore Joh. Aftbury. 8vo. Edin. 1781. p. 24.

9. Tentamen medicum inaugurale de variola. Auctore Joh. Ferriar. 8vo. Edin. 1781. p. 31.

10. Differtatio phyfica inauguralis de pubertate. Auctore Tho. Miller. 8vo. Edin. 1781. p. 35.

11. Differtatio medica inauguralis de afthmate fpafmodico. Auctore Jacobo Fenwick. 8vo. Edin. 1781. p. 16.

12. Tentamen phyfiologico-medicum inaugurale de perfpirationis ufu. Auctore Gulielmo Graham. 8vo. Edin. 1781. p. 46.

13. Tentamen medicum inaugurale de anafarca. Auctore Joh. Pleafance. 8vo. Edin. 1781. p. 52

14. Difputatio medica inauguralis de epilepfia. Auctore Joh. Waineman. 8vo. Edin. 1781. p. 25.

Ten-

15. Tentamen medicum inaugurale de epilepsia. Auctore Tipping Brown. 8vo. Edin. 1781. p. 52.

16. Dissertatio medica inauguralis de sale communi. Auctore Gulielm. Blackburne. 8vo. Edin. 1781. p. 36.

17. Tentamen medicum inaugurale de aëre, et imperio ejus in corpora humana. Auctore Edm. Cullen. 8vo. Edin. 1781. p. 45.

18. Disputatio medica inauguralis de dysenteriâ. Auctore Phil. Fletcher. 8vo. Edin. 1781.

19. Dissertatio medica inauguralis de colica Damnoniorum. Auctore Jac. Armistead. 8vo. Edin. 1781.

20. De la Philosophie; par M. *Beguin*, licentié en theologie, de la Societé Royale de Navarre, ancien professeur de philosophie en l'université de Paris. Tome troisiéme, où il est traité du son, de la lumiere, de l'odeur, de la saveur, et de l'electricité des corps naturels. *i. e.* Of Phisophy; by M. *Beguin*, licentiate in divinity, of the R. S. of Navarre, formerly professor of philosophy in the university of Paris. Vol. III. in which are treated of, sound, light, smell, taste, and the electricity of natural bodies. 8vo. Paris, 1780, 431 pages with copper-plates.

